# Supplementary material for: Deficiency of the frontotemporal dementia gene GRN results in gangliosidosis
Source: Nat Commun. 2022 Oct 7;13:5924. doi: 10.1038/s41467-022-33500-9 (PMC9546883; doi:10.1038/s41467-022-33500-9)
Supplement: Supplementary file 1 — Supplementary Information [file 41467_2022_33500_MOESM1_ESM.pdf]

Deficiency of the frontotemporal dementia gene *GRN* results in gangliosidosis

Sebastian Boland[1,2*], Sharan Swarup[2,3*], Yohannes A. Ambaw[1,2,4], Pedro C. Malia[1,2], Ruth C. Richards[1,2], Alexander W. Fischer[1,2], Shubham Singh[1,2], Geetika Aggarwal[5], Salvatore Spina[6], Alissa L. Nana[6], Lea T. Grinberg[6,7], William W. Seeley[6,7], Michal A. Surma[8], Christian Klose[8], Joao A. Paulo[2], Andrew D. Nguyen[5], J. Wade Harper[2,3], Tobias C. Walther[1,2,4,9,10*] and Robert V. Farese, Jr.[1,2,4,10*]

[1]Department of Molecular Metabolism, Harvard T. H. Chan School of Public Health, Boston, USA
[2]Department of Cell Biology, Harvard Medical School, Boston, MA 02115 USA
[3]Aligning Science Across Parkinson's (ASAP) Collaborative Research Network, Chevy Chase, MD 20815, USA
[4]Center on Causes and Prevention of Cardiovascular Disease, Harvard T. H. Chan School of Public Health, Boston, MA 02115 USA.
[5]Department of Internal Medicine, Division of Geriatric Medicine, and Department of Pharmacology and Physiology, Saint Louis University School of Medicine, St. Louis, MO 63104 USA
[6]Department of Neurology, Memory and Aging Center, University of California, San Francisco, San Francisco, CA 94158 USA
[7] Department of Pathology, University of California at San Francisco, San Francisco, USA
[8] Lipotype GmbH, Tatzberg 47, Dresden, Germany
[9] Howard Hughes Medical Institute, Boston, MA 02115, USA
[10] Broad Institute of Harvard and MIT, Cambridge, MA, 02124, USA

*The first authors (and last authors) contributed equally.

Corresponding authors:
J. Wade Harper (wade_harper@hms.harvard.edu), Tobias C. Walther (twalther@hsph.harvard.edu) and Robert V. Farese, Jr. (robert@hsph.harvard.edu)

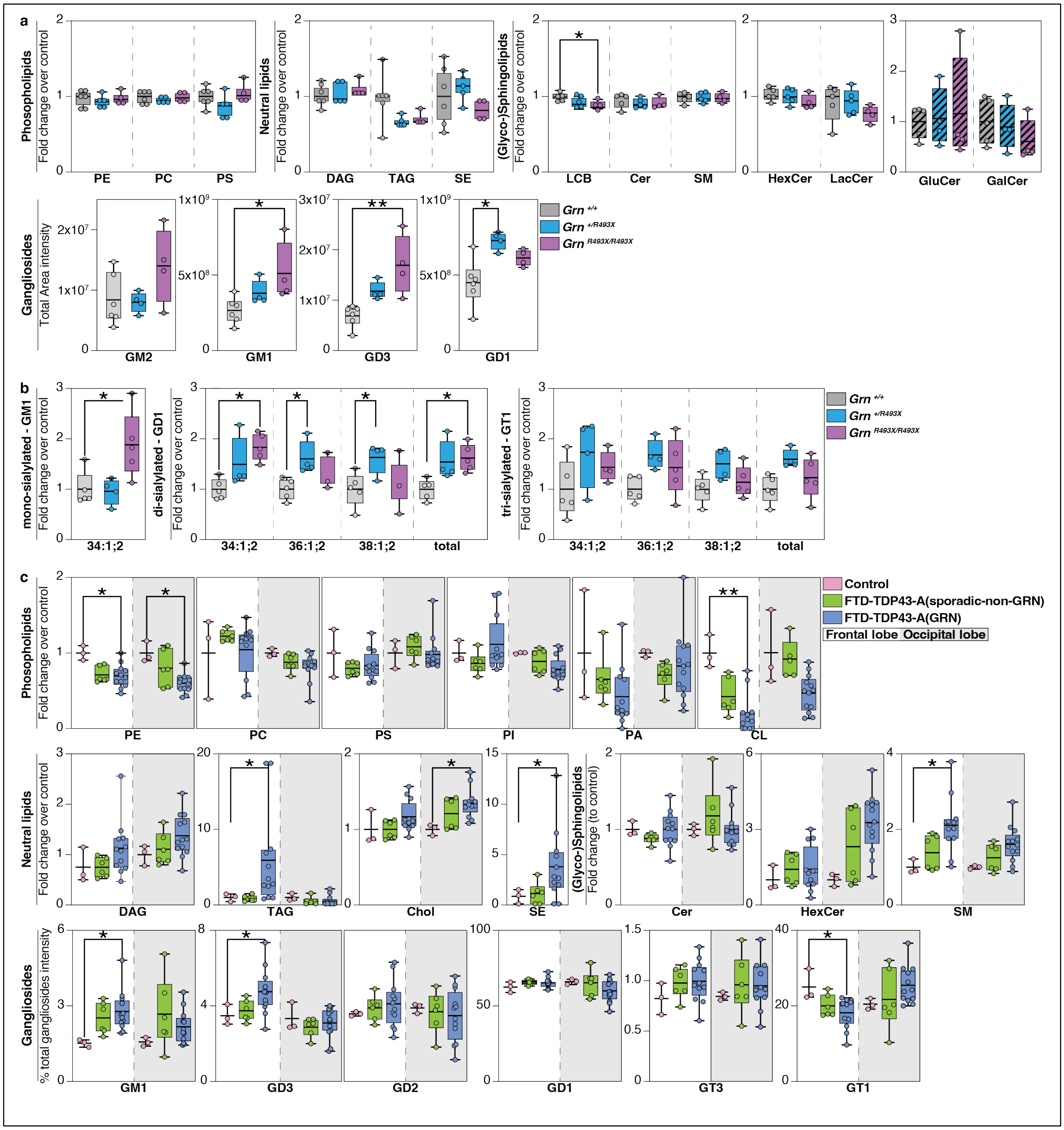

Supplementary Figure 1 | Global lipid analysis of mouse and human brains. a, Quantification of phospholipids (PE, PC, PS), neutral lipids (DAG, TAG, SE), glycosphingolipids (LCB, Cer, SM, HexCer, LacCer), and gangliosides (GM2, GM1, GD3, GD1) isolated from brains of *Grn* $^{+/+}$ (grey) (n=6), *Grn* $^{+/R493X}$ (blue) (n=5), and *Grn* $^{R493X/R493X}$ (purple) (n=*4)*. Quantification of GluCer and GalCer isolated from brains of *Grn* $^{+/+}$ (grey, diagonal stripes) (n=5), *Grn* $^{+/R493X}$ (blue, diagonal stripes) (n=4), and *Grn* $^{R493X/R493X}$ (purple, diagonal stripes) (n=4). **b,** Quantification of gangliosides (GM1, GD1, GT1) isolated from kideneys of *Grn* $^{+/+}$ (grey) (n=5), *Grn* $^{+/R493X}$ (blue) (n=4), and *Grn* $^{R493X/R493X}$ (purple) (n=*5*). **c**, Quantification of phospholipids (PE, PC, PS, PI, PA, CL), neutral lipids (DAG, TAG, Chol, SE), glyco-sphingolipids (Cer, HexCer, SM), and gangliosides (GM1, GD3, GD2, GD1, GT3, GT1) isolated

from the frontal and occipital lobes of control (pink) (n=3), FTD-TDP43-A (sporadic-non-GRN) (green) (n=6), and FTD-TDP43-A(GRN) (blue) (n=11 frontal, n=12 occipital) human brains. Box plots display mean ± the minimum and maximum number in the data set. One-way ANOVA, followed by multigroup comparison (Dunn's) test, was performed. *p<0.05, **p<0.01, or ***p<0.001. PE, phosphatidylthanolamine; PC, phosphatidylcholine; PS, phosphatidylserine; PI = phosphatidylinositol; PA, phosphatidic acid; CL, cardiolipin; DAG, diacylglycerol; TAG, triacylglycerol; Chol, cholesterol; SE, sterol-esters; Cer, ceramide; SM, sphingomyelin, HexCer, hexosylceramide; LacCer, lactosylceramide.

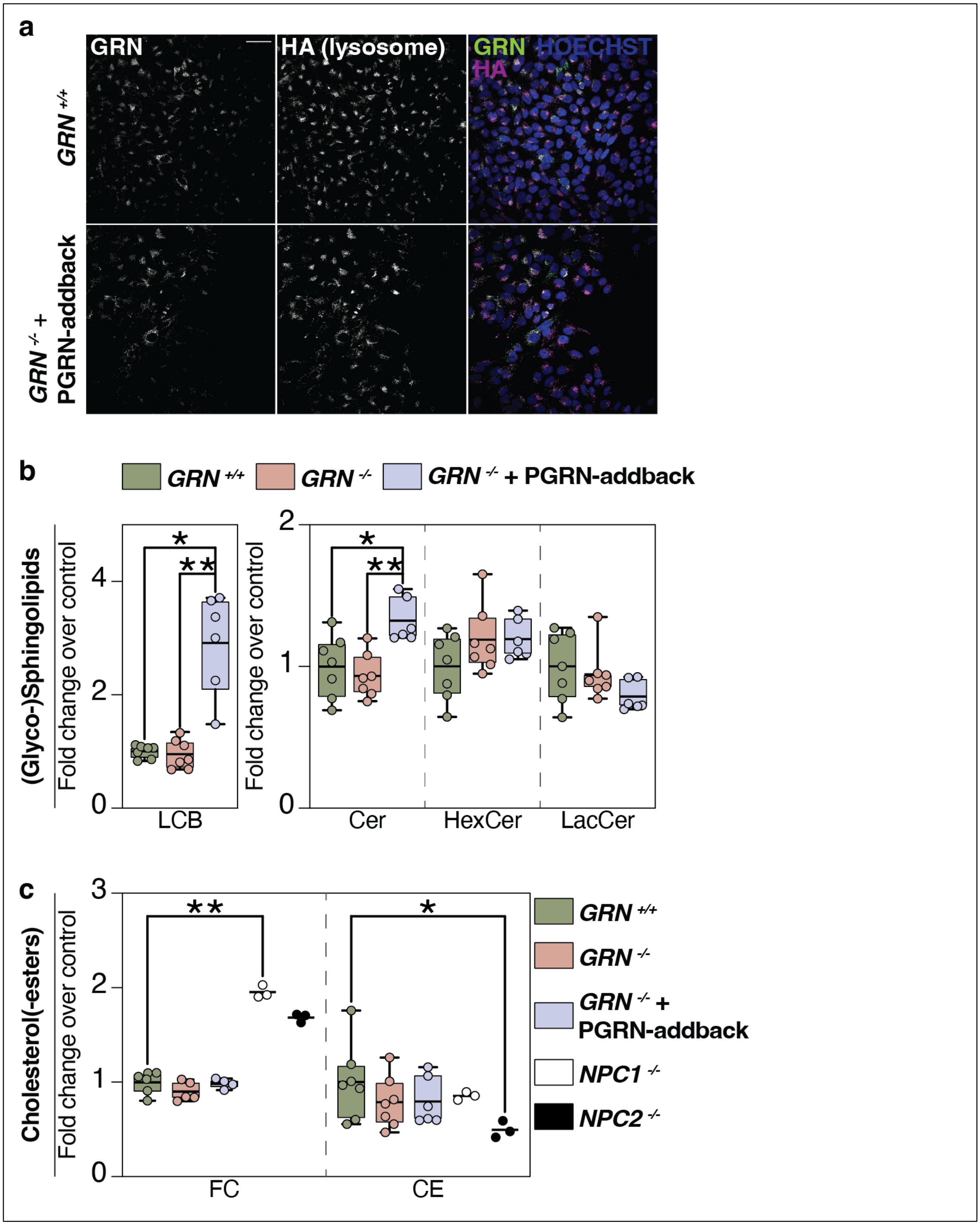

Supplementary Figure 2 | Loss of progranulin leads to GM2 accumulation in lysosomes. **a,** Representative confocal images of fixed GRN$^{+/+}$ and GRN$^{-/-}$ + PGRN-addback HeLa cells stained with anti-GRN antibody (green), anti-HA (lysosome) antibody (magenta) and Hoechst

(blue). Scale bar, 50 µm. **b,** Quantification of glycosphingolipids (LCB, Cer, HexCer, LacCer) isolated from *GRN*$^{+/+}$ (green) (n=7), *GRN*$^{-/-}$ (orange) (n=7), and *GRN*$^{-/-}$ + PGRN-addback (blue) (n=7) HeLa cell lines. **c**, Quantification of free cholesterol (FC) and cholesterol esters (CE) isolated from *GRN*$^{+/+}$ (green) (n=7), *GRN*$^{-/-}$ (orange) (n=7), and *GRN*$^{-/-}$ + PGRN-addback (blue) (n=7), NPC1$^{-/-}$ (white) (n=3), and NPC2$^{-/-}$ (black) (n=3) HeLa cell lines.

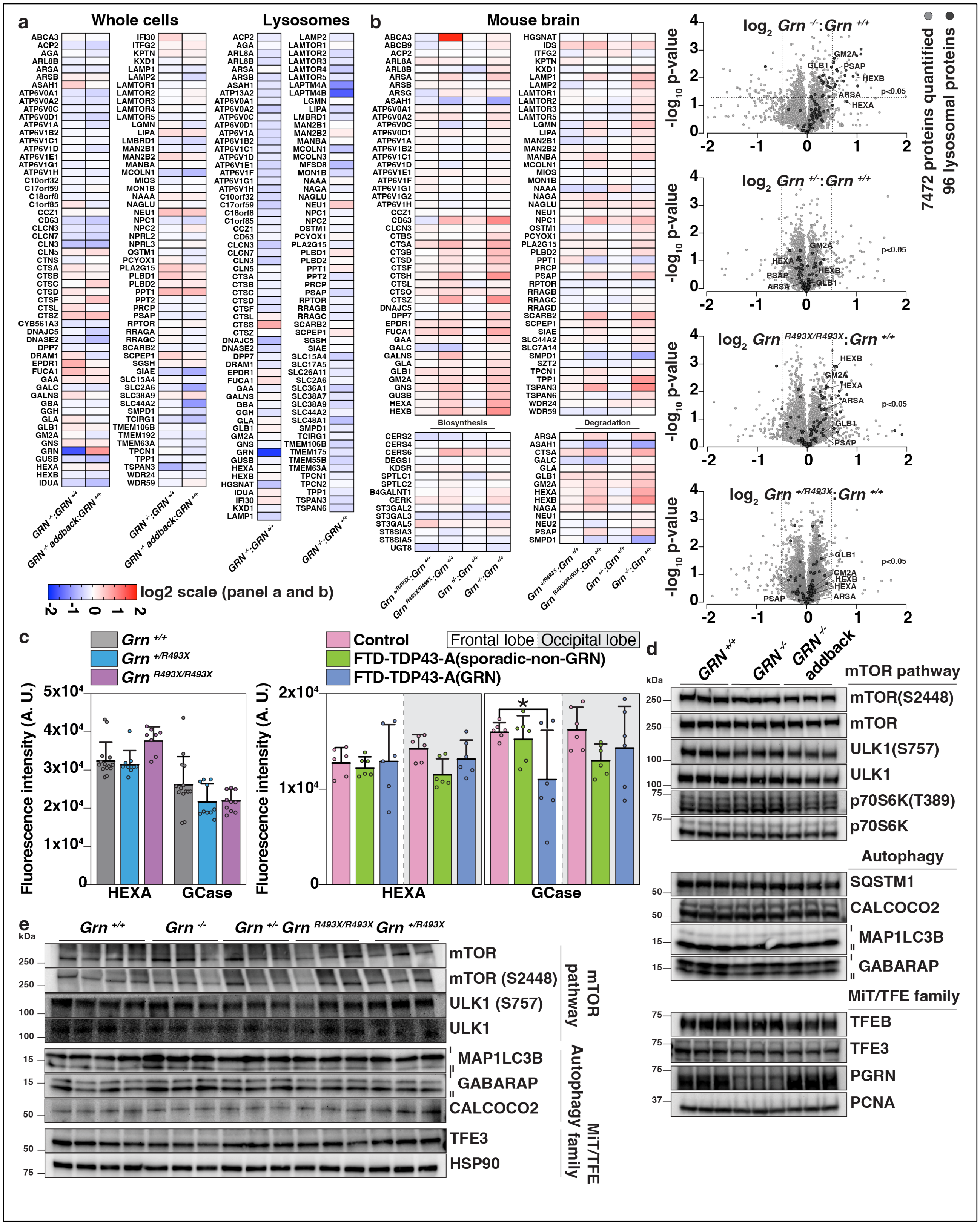

Supplementary Figure 3 | Quantitative TMT-proteomics, *in vitro* assays, and lysosomal functional assays of HeLa cell and mouse brains show no strong perturbations upon PGRN depletion. **a,** Heat-map representation of the relative abundance of lysosomal proteins

from HeLa whole-cell or lysosomes purified by Lyso-IP using the genotypes, *GRN*$^{+/+}$, *GRN*$^{-/-}$, and *GRN*$^{-/-}$ + PGRN-addback. **b,** Heat-map and volcano plot representation of the relative abundance of lysosomal proteins and glycosphingolipid metabolic enzymes from mouse brains of the following genotypes: *Grn* $^{+/+}$, *Grn* $^{-/-}$, *Grn* $^{+/-}$, *Grn* $^{+/R493X}$ and *Grn* $^{R493X/R493X}$. For the volcano plots, $\log_2$ fold-change (ratio of relative abundance) is on the x-axis and $-\log_{10}$ p-value on the y-axis. All proteins (grey) and lysosomal proteins (black) quantified are shown. A corrected p-value<0.05 (Welch's test, two-sided) was used to calculate significant differences between genotypes. **c**, HEXA and GCase activities were assessed in *Grn* $^{+/+}$, *Grn* $^{+/R493X}$ and *Grn* $^{R493X/R493X}$ mouse brain lysates (left) and in the frontal and occipital lobes of control (pink), FTD-TDP43-A(sporadic-non-GRN) (green), and FTD-TDP43-A(GRN) (blue) of human brain lysates as indicated using artificial substrates (n=3 with three technical replicates each, ±SD). One-way ANOVA, followed by multigroup comparison (Dunn's) test, was performed. *p<0.05, **p<0.01, or ***p<0.001. **d** and **e**, Western blotting analysis of phosphorylation of MiT/TFE family proteins (as measured by gel-shift assay of TFEB, TFE3), mTOR pathway activity (as measured by the phosphorylation of mTOR kinase and its substrates ULK1, p70S6K), and autophagic flux (as measured by lipidation of MAP1LC3B, GABARAP and levels of autophagy receptors SQSTM1, CALCOCO2) in HeLa whole-cell extracts (*GRN*$^{+/+}$, *GRN*$^{-/-}$, GRN$^{-/-}$ + PGRN-addback) and mouse brains (*Grn*$^{+/+}$, *Grn*$^{-/-}$, *Grn*$^{+/-}$, *Grn*$^{+/R493X}$,*Grn*$^{R493X/R493X}$).

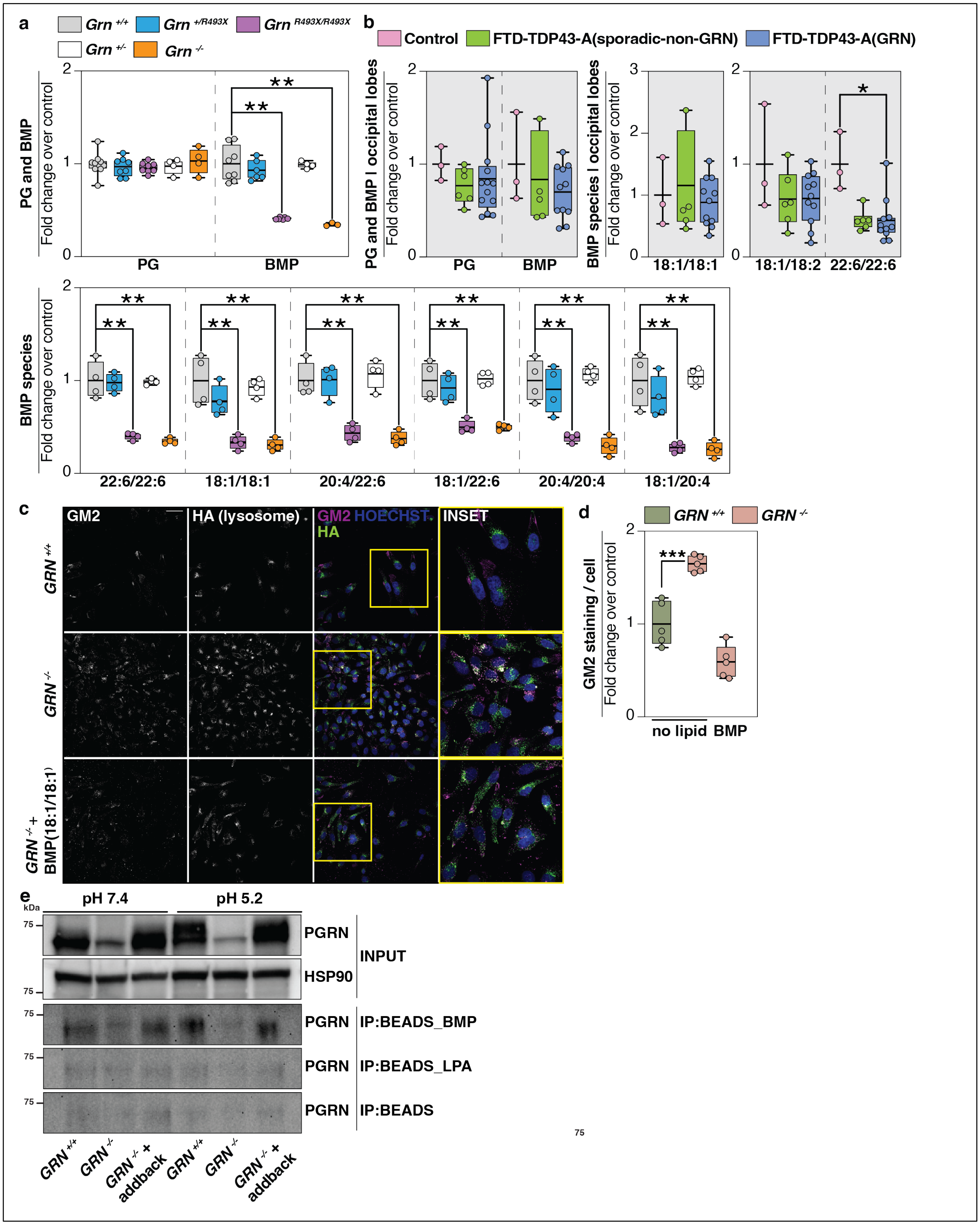
a
Grn +/+
Grn +/R493X
Grn R493X/R493X
Grn +/-
Grn -/-
PG and BMP
Fold change over control
PG
BMP
b
Control
FTD-TDP43-A(sporadic-non-GRN)
FTD-TDP43-A(GRN)
PG and BMP I occipital lobes
BMP species I occipital lobes
18:1/18:1
18:1/18:2
22:6/22:6
BMP species
22:6/22:6
18:1/18:1
20:4/22:6
18:1/22:6
20:4/20:4
18:1/20:4
c
GM2
HA (lysosome)
GM2 HOECHST HA
INSET
GRN +/+
GRN -/-
GRN -/- + BMP(18:1/18:1)
d
GRN +/+
GRN -/-
GM2 staining / cell
no lipid
BMP
e
kDa
pH 7.4
pH 5.2
PGRN
HSP90
INPUT
IP:BEADS_BMP
IP:BEADS_LPA
IP:BEADS
GRN +/+
GRN -/-
GRN -/- + addback

75

Supplementary Figure 4 | Loss of progranulin leads to reduced levels of BMP in cells, mouse brains, and human brains. **a,** Quantification of PG and BMP levels and individual BMP species from *Grn* $^{+/+}$ (grey) (n = 9), *Grn* $^{+/R493X}$ (blue) (n = 8), *Grn* $^{R493X/R493X}$ (purple) (n = 8), *Grn* $^{+/+}$ (white) (n = 4), and *Grn* $^{+/+}$ (orange) (n = 4) mouse brains. **b,** Quantification of PG and BMP levels and individual BMP species from the occipital lobes of control (pink) (n = 3), FTD-TDP43-A (sporadic-non-GRN) (green) (n = 6), and FTD-TDP43-A (GRN) (blue) (n = 12) human brains. Box plots display mean ± the minimum and maximum number in the data sets. One-way ANOVA, followed by multigroup comparison (Dunn's) test, was performed. *p<0.05, **p<0.01, or ***p<0.001. **c,** Representative immunofluorescent confocal image of fixed HeLa cell genotypes stained with anti-GM2 antibody (magenta), anti-HA (lysosome) antibody (green) and Hoechst (blue). Scale bar, 50 µm. **d,** Bar graphs display number of GM2 puncta per cell. One-way ANOVA, followed by multigroup comparison (Dunn's) test, was performed. ***p<0.001. **e,** Pull-down experiment of full-length PGRN using BMP-coated, LPA-coated, or control beads at pH 7.4 or pH 5.2 from *GRN*$^{+/+}$, *GRN*$^{-/-}$, and *GRN*$^{-/-}$ + PGRN-addback cell lysates. Western blotting analysis reveals binding of full-length endogenous and overexpressed PGRN to BMP-coated beads, in particular at pH 5.2.
