# Supplementary material for: Deficiency of the frontotemporal dementia gene GRN results in gangliosidosis
Source: Nat Commun. 2022 Oct 7;13:5924. doi: 10.1038/s41467-022-33500-9 (PMC9546883; doi:10.1038/s41467-022-33500-9)
Supplement: Supplementary file 3 — Description of Additional Supplementary Files [file 41467_2022_33500_MOESM3_ESM.pdf]

Description of Additional Supplementary Files

File name: Supplementary Data 1

Description: Comparative lipidomics analyses of mouse brain and kidney samples from $GRN^{+/+}$, $GRN^{+/R493X}$, $GRN^{R493X/R493X}$

File name: Supplementary Data 2

Description: Comparative lipidomics analyses of human brain samples from control, FTD-TDP43-A(sporadic-non-GRN), FTD-TDP43-A(GRN)

File name: Supplementary Data 3

Description: Comparative lipidomics analyses of HeLa cells from $GRN^{+/+}$, $GRN^{-/-}$, $GRN^{-/-}$ + addback

File name: Supplementary Data 4

Description: Quantitative proteomics analyses of HeLa cells from $GRN^{+/+}$, $GRN^{-/-}$, $GRN^{-/-}$ + addback

File name: Supplementary Data 5

Description: Quantitative proteomics analyses of HeLa lysosomes from $GRN^{+/+}$, $GRN^{-/-}$

File name: Supplementary Data 6

Description: Quantitative proteomics analyses of mouse brain samples from $GRN^{+/+}$, $GRN^{+/R493X}$, $GRN^{R493X/R493X}$,$GRN^{+/-}$, $GRN^{-/-}$

File name: Supplementary Data 7

Description: Genotype, sex, and clinical description of human brain samples
