# Supplementary material for: Deficiency of the frontotemporal dementia gene GRN results in gangliosidosis
Source: Nat Commun. 2022 Oct 7;13:5924. doi: 10.1038/s41467-022-33500-9 (PMC9546883; doi:10.1038/s41467-022-33500-9)
Supplement: Supplementary file 11 — Reporting Summary [file 41467_2022_33500_MOESM11_ESM.pdf]

nature portfolio

Corresponding author(s): Robert Farese, Jr.

Last updated by author(s): July 16, 2022

Reporting Summary

Nature Portfolio wishes to improve the reproducibility of the work that we publish. This form provides structure for consistency and transparency in reporting. For further information on Nature Portfolio policies, see our Editorial Policies and the Editorial Policy Checklist.

Statistics

For all statistical analyses, confirm that the following items are present in the figure legend, table legend, main text, or Methods section.

n/a	Confirmed	
☐	☒	The exact sample size (*n*) for each experimental group/condition, given as a discrete number and unit of measurement
☐	☒	A statement on whether measurements were taken from distinct samples or whether the same sample was measured repeatedly
☐	☒	The statistical test(s) used AND whether they are one- or two-sided<br>*Only common tests should be described solely by name; describe more complex techniques in the Methods section.*
☒	☐	A description of all covariates tested
☒	☐	A description of any assumptions or corrections, such as tests of normality and adjustment for multiple comparisons
☐	☒	A full description of the statistical parameters including central tendency (e.g. means) or other basic estimates (e.g. regression coefficient) AND variation (e.g. standard deviation) or associated estimates of uncertainty (e.g. confidence intervals)
☐	X	For null hypothesis testing, the test statistic (e.g. *F*, *t*, *r*) with confidence intervals, effect sizes, degrees of freedom and *P* value noted<br>*Give P values as exact values whenever suitable.*
X	☐	For Bayesian analysis, information on the choice of priors and Markov chain Monte Carlo settings
X	☐	For hierarchical and complex designs, identification of the appropriate level for tests and full reporting of outcomes
☐	X	Estimates of effect sizes (e.g. Cohen's *d*, Pearson's *r*), indicating how they were calculated

Our web collection on statistics for biologists contains articles on many of the points above.

Software and code

Policy information about availability of computer code

Data collection	No software was used for data collection in this study.
Data analysis	Lipid Search (version 4.1), GraphPad Prism (version 9.0)

For manuscripts utilizing custom algorithms or software that are central to the research but not yet described in published literature, software must be made available to editors and reviewers. We strongly encourage code deposition in a community repository (e.g. GitHub). See the Nature Portfolio guidelines for submitting code & software for further information.

Data

Policy information about availability of data

All manuscripts must include a data availability statement. This statement should provide the following information, where applicable:

- Accession codes, unique identifiers, or web links for publicly available datasets
- A description of any restrictions on data availability
- For clinical datasets or third party data, please ensure that the statement adheres to our policy

No restrictions on data availability. The mass spectrometry lipidomics and proteomics raw data generated in this study have been deposited in the ProteomeXchange Consortium via the PRIDE partner repository under accession code identifier 1PXD029551.

Field-specific reporting

Please select the one below that is the best fit for your research. If you are not sure, read the appropriate sections before making your selection.

☒ Life sciences ☐ Behavioural & social sciences ☐ Ecological, evolutionary & environmental sciences

For a reference copy of the document with all sections, see nature.com/documents/nr-reporting-summary-flat.pdf

Life sciences study design

All studies must disclose on these points even when the disclosure is negative.

Sample size The sample sizes used for every experiment were determined based on the availability of samples, experimental technical logistics, and statistical criteria.

Data exclusions No data was excluded from the analyses performed for this study.

Replication >3 independent biological replicates were used for all lipidomics, proteomics, and cellular assays used in this study to confirm the reproducibility of the experimental findings.

Randomization The treatment of samples, the collection of samples, the processing of samples, and their analyses were randomized in all experiments involving cell lines or mouse models.

Blinding The investigators were not blinded while performing experiments for this study.

Reporting for specific materials, systems and methods

We require information from authors about some types of materials, experimental systems and methods used in many studies. Here, indicate whether each material, system or method listed is relevant to your study. If you are not sure if a list item applies to your research, read the appropriate section before selecting a response.

Materials & experimental systems

n/a	Involved in the study	
☐	☒	Antibodies
☐	☒	Eukaryotic cell lines
☒	☐	Palaeontology and archaeology
☐	☒	Animals and other organisms
☐	☒	Human research participants
☒	☐	Dual use research of concern

Methods

n/a	Involved in the study	
☒	☐	ChIP-seq
☒	☐	Flow cytometry
☒	☐	MRI-based neuroimaging

Antibodies

Antibodies used GM2 monoclonal antibody (TCI America, clone MK1-16 Cat#A2576), human progranulin (R&D systems, Cat#AF2420), PCNA (Santa Cruz Biotechnology Cat#sc56), GALC (Proteintech Cat#11991-1-AP), GLA (Proteintech Cat#66121-1-lg), GM2A (Proteintech Cat#10864-2-AP), NEUl (Santa Cruz Biotechnology Cat#sc166824), PSAP (Proteintech Cat#10801-1-AP), GLBl (Proteintech Cat#15518-1-AP), HEXA (Proteintech Cat#11317-1-AP), HSP90 (Proteintech Cat#60318-1-lg), TFEB (Cell Signaling Technology Cat#4240), TFE3 (Proteintech Cat#14480-1-AP), SQSTMl (Proteintech Cat#18420-1-AP), CALCOC02 (Proteintech Cat#12229-1-AP), MAP1LC3B (Cell Signaling Technology Cat#2775), GABARAP (Proteintech Cat#18723-1-AP), MTOR (Cell Signaling Technology Cat#2983), MTOR (52448) (Cell Signaling Technology Cat#2971), P7056K (Cell Signaling Technology Cat#2708), P7056K (T389) (Cell Signaling Technology Cat#9234), ULKl (Cell Signaling Technology Cat#8054), ULKl (5757) (Cell Signaling Technology Cat#14202), TMEM106B (Bethyl Labs Cat#A303439A-M), ASAHl (Proteintech Cat#11274-1-AP), HEXB (Proteintech Cat#16229-1-AP), BETA-ACTIN (Santa Cruz Biotechnology Cat#69879), and Alexa Fluor (Thermo Fisher Cat#A32731 Cat#21203).

Validation All the antibodies used for immunoblotting and immunofluorescence have been previously used in publications and /or validated by manufacturer (refer to website).

Eukaryotic cell lines

Policy information about cell lines

Cell line source(s) American Type Culture Collection (ATCC)

Authentication HeLa (Cat#CCL-2) and HEK293T (Cat#CRL-3216) purchased from ATCC (authentication, karyotyping performed by ATCC).

Mycoplasma contamination	All cell lines used in this study tested negative for mycoplasma contamination
Commonly misidentified lines (See ICLAC register)	No commonly misidentified lines were used in this study.

Animals and other organisms

Policy information about studies involving animals; ARRIVE guidelines recommended for reporting animal research

Laboratory animals	Mice carrying a humanized nonsense mutation R493X in mouse Grn gene (GrnR493X)(Grntm2.1Far/J, JAX#029919) were previously generated in our laboratory. Tissues were collected from mice of both sexes at 18-19 months of age. Mouse husbandry conditions, including ambient temperature, humidity and dark/light cycle followed the guidelines established by the Harvard Center for Comparative Medicine. Mice were maintained in a barrier facility, at normal room temperatures, on a regular 12-h light and 12-h dark cycle and had ad libitum access to food and water unless otherwise stated.
Wild animals	No field wild animals were used in this study.
Field-collected samples	No field collected samples were used in this study.
Ethics oversight	All experiments were conducted in accordance with the Harvard Medical Area Standing Committee on Animals (IACUC Protocol# 05121) and followed NIH guidelines.

Note that full information on the approval of the study protocol must also be provided in the manuscript.
